# Comparison of the Effects of Dexmedetomidine Versus Lidocaine in Blunting the Hemodynamic Response to Tracheal Extubation in Patients Undergoing Craniotomy

**DOI:** 10.7759/cureus.107503

**Published:** 2026-04-21

**Authors:** Umama Masnoon, Maria Hashmi, Hubba Ahmed, Tooba Usman, Alina Mahmood, Sana Urooj

**Affiliations:** 1 Department of Anesthesiology, Dow International Medical College (DIMC), Ojha Campus, Karachi, PAK; 2 Department of Anesthesia, Aga Khan University Hospital, Karachi, PAK; 3 Department of Anesthesiology, Our Lady of Lourdes Hospital, Drogheda, IRL

**Keywords:** craniotomy, dexmedetomidine, hemodynamic response, lidocaine, tracheal extubation

## Abstract

Background

Tracheal extubation following neurosurgical procedures is commonly associated with sympathetic stimulation, leading to tachycardia and hypertension. Such hemodynamic fluctuations may increase intracranial pressure (ICP) and adversely affect cerebral perfusion in patients undergoing craniotomy. Various pharmacological agents have been used to attenuate these responses; however, the comparative effectiveness of dexmedetomidine and lidocaine during tracheal extubation remains an area of clinical interest.

Objectives

The objective of this study is to compare the effectiveness of dexmedetomidine and lidocaine in attenuating the hemodynamic response to tracheal extubation in patients undergoing craniotomy.

Study design, setting, and methodology

This was a prospective comparative study conducted in the neurosurgery operating theatres of Civil Hospital Karachi, Karachi, Pakistan.

A total of 60 patients undergoing craniotomy were included and divided into two groups (n = 30 each). Group A received intravenous dexmedetomidine (0.5 µg/kg), and Group B received intravenous lidocaine (1.5 mg/kg) prior to extubation. Hemodynamic parameters, including heart rate (HR), systolic blood pressure (SBP), diastolic blood pressure (DBP), and mean arterial pressure (MAP), were recorded at baseline and at 0, 1, 3, 5, and 10 minutes after extubation. Postoperative pain score, sedation score, and extubation quality were also assessed. Data were analyzed using IBM SPSS Statistics for Windows, Version 22 (Released 2013; IBM Corp., Armonk, NY, USA), and a p-value ≤ 0.05 was considered statistically significant.

Results

Baseline demographic characteristics were comparable between the two groups. Hemodynamic parameters remained similar during the early post-extubation period; however, significant increases in HR, SBP, DBP, and MAP were observed in the lidocaine group at 5 and 10 minutes after extubation (p < 0.05). Additionally, the dexmedetomidine group demonstrated significantly lower pain scores (1.57 ± 0.62 vs 3.07 ± 1.98), better extubation quality (1.00 ± 0.78 vs 2.10 ± 0.84), and lower sedation scores (1.43 ± 0.56 vs 2.17 ± 0.69), compared with the lidocaine group (p = 0.0005).

Conclusion

Dexmedetomidine was more effective than lidocaine in attenuating the hemodynamic response to tracheal extubation and provided better postoperative recovery outcomes in patients undergoing craniotomy.

## Introduction

Patients undergoing neurosurgical techniques, especially craniotomy, have significant concern about the diminution of the creative hemodynamic response during tracheal extubation [1]. Intracranial pressure (ICP) and cerebral blood flow can spike suddenly after extubation due to sympathetic activation, which causes tachycardia and hypertension [2]. Such fluctuations may compromise cerebral perfusion pressure and increase the risk of cerebral edema or hemorrhage, especially in those with impaired cerebral autoregulation [3].

Craniotomy procedures involve significant surgical stimulation and require an anesthetic technique that provides both hemodynamic stability and rapid recovery from sedation. Because of the critical importance of early postoperative neurological evaluation in neurosurgical patients, controlled and smooth extubation is a key component of anesthetic management [4]. However, tracheal extubation is frequently associated with airway irritation and activation of the sympathetic nervous system, which causes an abrupt increase in heart rate (HR) and blood pressure. Postoperative pain, emergence from anesthesia, catecholamine release, and airway stimulation are the primary mediators of these responses. Although these hemodynamic changes are often transient, they can have serious consequences in neurosurgical patients if not adequately controlled [5].

Hemodynamic management becomes even more challenging in patients with preexisting hypertension or other comorbidities undergoing neurosurgical procedures. A number of pharmacological agents, such as opioids, propofol, beta-blockers, and α2-adrenergic agonists, have been employed to reduce the intensity of these reactions [6]. Opioids are commonly used to suppress sympathetic responses during anesthesia; however, excessive opioid administration may lead to respiratory depression and carbon dioxide retention, which can increase ICP during recovery. Similarly, clonidine and other alpha-2 agonists have been utilized to reduce hypertensive reactions; nevertheless, they have the potential to induce bradycardia and hypotension, especially in older patients [7]. In neuroanesthesia, any pharmacological intervention must not only stabilize hemodynamics but also preserve intracranial homeostasis and allow rapid neurological assessment following surgery. An ideal drug should maintain stable cerebral perfusion, reduce sympathetic stimulation, and avoid respiratory depression [8].

Dexmedetomidine, an anesthetic adjuvant, has recently attracted considerable interest due to its high selectivity as an α2-adrenergic receptor agonist. Without significantly increasing respiratory depression, it causes dose-dependent sedation, analgesia, and anxiolysis. Dexmedetomidine has been shown to provide stable hemodynamics during the perioperative period and reduce intraoperative opioid requirements. Moreover, experimental studies have suggested that dexmedetomidine may possess neuroprotective properties, making it particularly useful in neurosurgical anesthesia [9].

Lidocaine (lignocaine), an amide local anesthetic, has also been widely used to blunt the hemodynamic response associated with tracheal stimulation. It can be administered intravenously or applied topically to the airway to reduce coughing, suppress airway reflexes, and attenuate sympathetic responses during intubation and extubation. In addition to lowering airway responsiveness by reducing intracellular calcium in airway smooth muscle, lidocaine has been found to decrease increases in ICP during tracheal suctioning [10].

Research has shown that both groups experience variations in HR, diastolic blood pressure (DBP), and systolic blood pressure (SBP) following extubation; however, dexmedetomidine significantly mitigates these changes. Additionally, dexmedetomidine has been associated with improved sedation, reduced coughing, and better extubation quality compared with lidocaine [11,12].

Despite these findings, limited local and regional data are available regarding the comparative effectiveness of lidocaine and dexmedetomidine in reducing hemodynamic responses during extubation in neurosurgical patients. Thus, the purpose of this study was to evaluate how effectively lidocaine and dexmedetomidine reduce the hemodynamic response to tracheal extubation in craniotomy patients. The main objective was to compare the hemodynamic responses between the two drugs. Secondary objectives included evaluating postoperative sedation levels and the quality of extubation. It was hypothesized that dexmedetomidine would outperform lidocaine in reducing the hemodynamic response to tracheal extubation in craniotomy patients.

## Materials and methods

The research took place in elective and emergency neurosurgery operating rooms at Civil Hospital Karachi, Karachi, Pakistan. The investigation lasted six months, from June 2025 until December 2025. After obtaining permission from the Institutional Review Board (Ref No. IRB-3712/DUHS/Approval/2025/387, dated June 6, 2025), the study was initiated. Using the OpenEpi program, we determined the sample size by calculating the 95% confidence interval and 80% study power. In the dexmedetomidine group, the mean ± standard deviation of HR at zero minutes after extubation was 94.36 ± 12.19, while in the lidocaine group it was 110.46 ± 14.91, based on previous research [12]. A total of 60 participants were included in the study, with 30 patients allocated to each group. A non-probability consecutive sampling technique was used. As this was a non-randomized study, there was a potential risk of selection bias; however, efforts were made to minimize this by applying strict inclusion and exclusion criteria and ensuring comparable baseline characteristics between the two groups.

Patients undergoing craniotomy ranged in age from 18 to 60 and were classified as having a physical status of I or II by the American Society of Anesthesiologists (ASA). Pregnant females, patients with bradycardia or hypotension, and patients with significant systemic diseases such as chronic hypertension were excluded. These included conditions such as congestive heart failure, congenital heart defects, arrhythmias, dysfunction of the endocrine or autonomic systems, airway diseases (restrictive or obstructive), and disorders affecting the liver, kidneys, or metabolism.

Patients receiving antihypertensive or antiarrhythmic medications, those with a history of intolerance to beta-blockers, narcotic dependence, known drug allergies, and those requiring postoperative ventilatory support were also excluded to ensure patient safety and minimize confounding factors that could influence hemodynamic responses.

Prior to enrollment, all patients were requested to sign informed consent after a comprehensive explanation of the study’s purpose, methodology, risks, and benefits. Two groups, Group A and Group B, were formed from the eligible patients. Individuals in Group A were administered 0.5 µg/kg of dexmedetomidine diluted in 100 mL of 0.9% normal saline intravenously, whereas those in Group B were administered 1.5 mg/kg of lidocaine diluted in 100 mL of 0.9% normal saline. The study drugs were prepared by the principal investigator or co-investigator and handed over to the primary anesthesiologist, who remained blinded to the study protocol.

Once the patient was moved to the operating area, an 18-gauge intravenous catheter was placed, and baseline measurements were collected using non-invasive blood pressure (NIBP) monitoring and pulse oximetry to record vital signs, including HR, peripheral oxygen saturation, and electrocardiogram (ECG). Every patient received an intravenous injection of 2 mg/kg propofol, 0.1 mg/kg nalbuphine, and 0.5 mg/kg atracurium for induction of anesthesia. The airway was secured after ensuring adequate muscle relaxation.

An invasive arterial line was inserted after induction for further monitoring. Maintenance of anesthesia was achieved using isoflurane and an atracurium infusion. Atracurium infusion was discontinued after dural closure. At the time of surgical dressing, the primary anesthesiologist administered the study drug over 10 minutes after recording baseline HR, NIBP, and SpO₂.

Neostigmine (0.05 mg/kg) and glycopyrrolate (0.2 mg) were administered to reverse neuromuscular blockade once isoflurane was discontinued. The patient’s oropharyngeal secretions were suctioned before extubation. Once extubation criteria were met, the endotracheal tube was removed.

Hemodynamic parameters, including HR, SBP, DBP, and mean arterial pressure (MAP), were recorded at baseline and at 0, 1, 3, 5, and 10 minutes after extubation. The study drugs were administered intravenously 10 minutes prior to planned extubation. In addition, sedation score, extubation quality, and pain score were assessed at the time of extubation. Any hemodynamic changes were managed with appropriate medications and recorded along with any adverse effects.

The patient was sent to the recovery room after extubation, and the post-anesthesia care unit assessed their level of sedation at zero minutes using the Revised Ramsay Sedation Scale [13]. We used IBM SPSS Statistics for Windows, Version 22 (Released 2013; IBM Corp., Armonk, NY, USA) to perform the analysis. All research variables had descriptive statistics computed. Statistical measures such as mean ± standard deviation were calculated for continuous variables. Categorical data were analyzed using frequencies and percentages. The results of the study were compared between the two groups using an independent-samples t-test, with a p-value of ≤ 0.05 considered statistically significant. 

Operational definitions

Hemodynamic Response

Hemodynamic response was assessed by measuring blood pressure readings taken at the moment of extubation (zero minutes): MAP, SBP, DBP, and HR.

Extubation Quality

Extubation quality was evaluated by grading the severity of cough using a standardized cough grading scale.

Sedation Score

Sedation was assessed at zero minutes after extubation using the Ramsay Sedation Scale, where lower scores indicate a higher level of alertness (1 = anxious/agitated and 2 = cooperative and oriented), and higher scores indicate deeper sedation [13].

Pain Score

The Visual Analog Scale (VAS) was used to measure the intensity of pain on a scale from 0 to 10, where 0 signifies no pain and 10 signifies the worst imaginable pain [13].

## Results

The mean age of patients in Group A and Group B was 42.77 ± 8.01 years and 43.47 ± 9.94 years, respectively, indicating no substantial difference between the groups. In Group A, 23 (38.33%) patients were male and seven (11.67%) were female, whereas Group B included 17 (28.33%) males and 13 (21.67%) females. Regarding ASA status, 17 (28.33%) patients in Group A and 16 (26.67%) patients in Group B were classified as ASA-I, while ASA-II patients accounted for 13 (21.67%) and 14 (23.33%) in Group A and Group B, respectively. The mean body weight in Group A and Group B was 67.43 ± 9.03 kg and 67.30 ± 8.56 kg, respectively. Similarly, the mean height was 154.40 ± 5.09 cm in Group A and 155.20 ± 5.81 cm in Group B. The mean body mass index (BMI) was 28.27 ± 3.38 kg/m² in Group A and 27.93 ± 3.01 kg/m² in Group B. Overall, these findings indicate that both groups were comparable at baseline with respect to demographic and anthropometric characteristics, as given in Table 1.

**Table 1:** Comparison of baseline demographic and anthropometric characteristics between the study groups

Variables	Group A (n = 30), Mean ± SD	Group B (n = 30), Mean ± SD
Age (Years)	42.77 ± 8.01	43.47 ± 9.94
Sex		
Male	23 (38.33%)	17 (28.33%)
Female	7 (11.67%)	13 (21.67%)
ASA Status		
ASA-I	17 (28.33%)	16 (26.67%)
ASA-II	13 (21.67%)	14 (23.33%)
Weight (kg)	67.43 ± 9.03	67.30 ± 8.56
Height (cm)	154.40 ± 5.09	155.20 ± 5.81
BMI (kg/m²)	28.27 ± 3.38	27.93 ± 3.01

The operative characteristics showed differences between the two study groups. The mean length of anesthesia in Group A was 95.43 ± 7.35 minutes compared with 111.73 ± 9.68 minutes in Group B. Similarly, the mean duration of surgery was 89.63 ± 6.64 minutes in Group A and 102.30 ± 6.43 minutes in Group B. Furthermore, the mean extubation time was shorter in Group A (15.40 ± 1.32 minutes) compared with Group B (18.23 ± 3.11 minutes). Overall, patients in Group A demonstrated relatively shorter anesthesia duration, surgical time, and extubation time compared with those in Group B, as given in Table 2.

**Table 2:** Comparison of intraoperative and extubation characteristics between the study groups

Variables	Group A (n = 30), Mean ± SD	Group B (n = 30), Mean ± SD
Length of Anesthesia (min)	95.43 ± 7.35	111.73 ± 9.68
Length of Surgery (min)	89.63 ± 6.64	102.30 ± 6.43
Extubation Time (min)	15.40 ± 1.32	18.23 ± 3.11

The mean pain score was significantly lower in Group A compared with Group B (1.57 ± 0.62 vs 3.07 ± 1.98; p = 0.0005). Similarly, the extubation quality score was significantly better in Group A than in Group B (1.00 ± 0.78 vs 2.10 ± 0.84; p = 0.0005). In addition, the mean sedation score was significantly lower in Group A compared with Group B (1.43 ± 0.56 vs 2.17 ± 0.69; p = 0.0005), indicating improved postoperative recovery in patients receiving dexmedetomidine, as given in Table 3.

**Table 3:** Comparison of mean pain score, extubation quality and sedation score between groups Data are presented as mean ± standard deviation. Comparison between Group A and Group B was performed using the independent sample t-test. Test statistics are reported as t(58), where 58 represents the degrees of freedom (df = n₁ + n₂ - 2). A p-value ≤ 0.05 was considered statistically significant.

Outcome Variable	Group A (n = 30), Mean ± SD	Group B (n = 30), Mean ± SD	t-value (df = 58)	p-value
Pain Score	1.57 ± 0.62	3.07 ± 1.98	3.96	0.0005
Extubation Quality	1.00 ± 0.78	2.10 ± 0.84	5.26	0.0005
Sedation Score	1.43 ± 0.56	2.17 ± 0.69	4.57	0.0005

**Figure 1:**
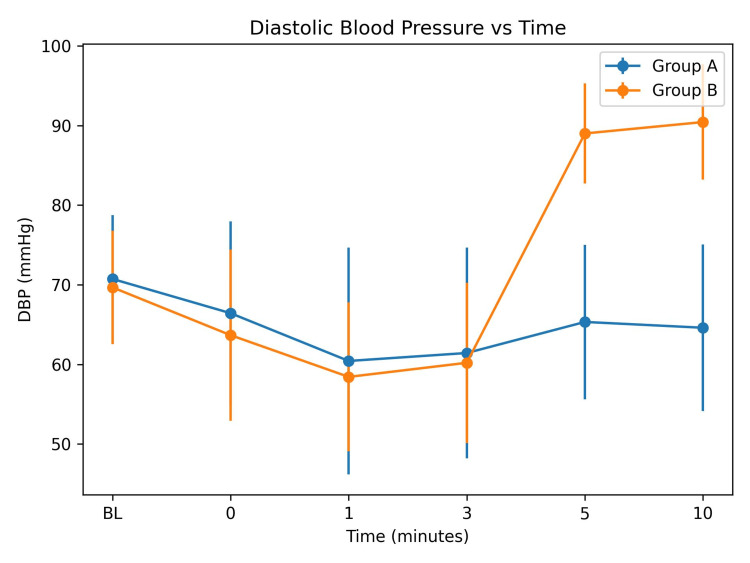
Comparison of diastolic blood pressure (DBP) between Group A and Group B at different time intervals after extubation (mean ± SD)

DBP was comparable between both groups at baseline (70.73 ± 8.00 vs 69.67 ± 7.12 mmHg; p = 0.587) and during the early post-extubation period (0-3 minutes), with no statistically significant difference (p > 0.05). However, DBP increased significantly in Group B at five minutes (89.00 ± 6.29 vs 65.33 ± 9.70 mmHg; p = 0.0005) and 10 minutes (90.43 ± 7.24 vs 64.60 ± 10.44 mmHg; p = 0.0005) compared with Group A (Figure 1).

**Figure 2:**
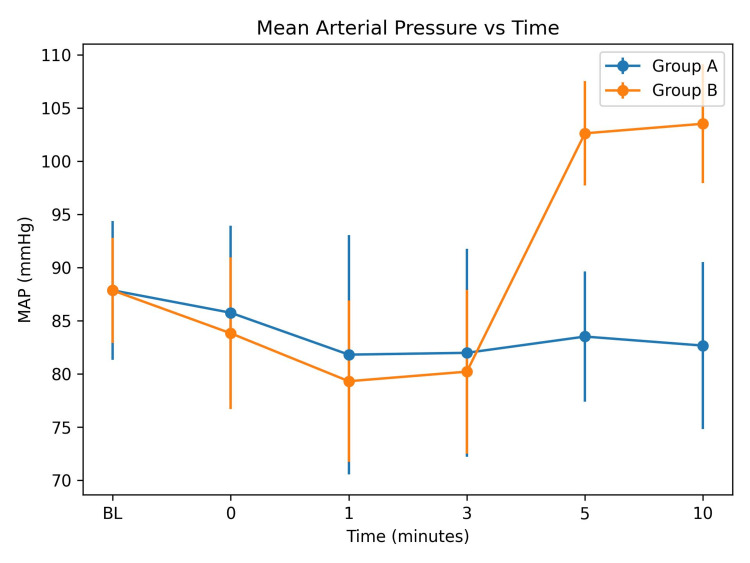
Comparison of mean arterial pressure (MAP) between Group A and Group B at different time intervals after extubation (mean ± SD)

MAP was similar between the two groups at baseline (87.87 ± 6.53 vs 87.86 ± 4.96 mmHg; p = 0.994) and during the early post-extubation period (0-3 minutes; p > 0.05). At five minutes, MAP was significantly higher in Group B compared with Group A (102.63 ± 4.90 vs 83.52 ± 6.13 mmHg; p = 0.0005), and the difference remained significant at 10 minutes (103.53 ± 5.58 vs 82.67 ± 7.86 mmHg; p = 0.0005), as shown in Figure 2.

**Figure 3:**
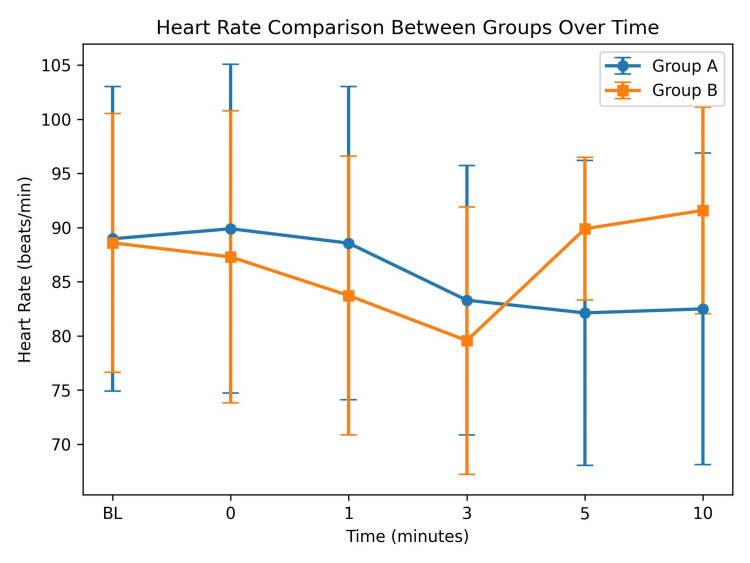
Comparison of heart rate (HR) between Group A and Group B at different time intervals after extubation (mean ± SD)

HR was comparable between the groups at baseline (88.97 ± 14.07 vs 88.60 ± 11.95 beats/min; p = 0.941) and up to three minutes after extubation (p > 0.05). However, HR increased significantly in Group B at five minutes (89.90 ± 6.58 vs 82.13 ± 14.09 beats/min; p = 0.008) and 10 minutes (91.60 ± 9.53 vs 82.50 ± 14.39 beats/min; p = 0.005) compared with Group A, as shown in Figure 3.

**Figure 4:**
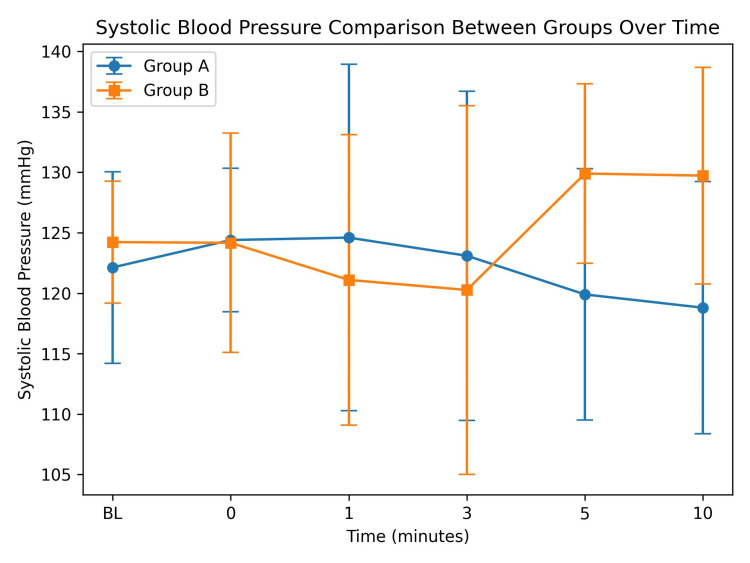
Comparison of systolic blood pressure (SBP) between Group A and Group B at different time intervals after extubation (mean ± SD)

SBP showed no significant difference between the two groups at baseline (122.13 ± 7.92 vs 124.23 ± 5.04 mmHg; p = 0.226) and during the early post-extubation period (0-3 minutes; p > 0.05). However, SBP was significantly higher in Group B at five minutes (129.90 ± 7.41 vs 119.90 ± 10.40 mmHg; p = 0.0005) and 10 minutes (129.73 ± 8.97 vs 118.80 ± 10.44 mmHg; p = 0.0005), as shown in Figure 4.

## Discussion

Tracheal extubation following neurosurgical procedures is frequently linked to substantial hemodynamic reactions, including rapid HR and high blood pressure, as a result of sympathetic activation. Patients undergoing craniotomy may experience unfavourable neurological outcomes due to altered cerebral perfusion and elevated ICP. To lessen these reactions during extubation, a number of pharmacological agents have been employed. Dexmedetomidine and lidocaine are commonly used agents; however, their comparative effectiveness in blunting hemodynamic responses during tracheal extubation remains an area of clinical interest. Attenuation of the hemodynamic response during tracheal extubation is an important objective in neuroanesthesia, particularly in patients undergoing craniotomy, where unwanted spikes in ICP and impaired cerebral perfusion can result from rapid elevations in HR and blood pressure. Dexmedetomidine produced considerably greater hemodynamic stability and improved postoperative recovery outcomes compared with lidocaine, according to the current study, which examined the effects of the two drugs on attenuation of the hemodynamic response during tracheal extubation [14].

Our results align with those of Kothari et al. (2014), who found that dexmedetomidine and lignocaine both reduced hemodynamic responses during extubation. Interestingly, the dexmedetomidine group showed a significantly lower rise in HR, SBP, as well as DBP (p < 0.05) [15]. Dexmedetomidine during extubation was associated with substantially reduced HR and blood pressure levels compared with lignocaine, according to Munir et al. (2025). At extubation, the dexmedetomidine group had a HR of 89.40 beats per minute, while the lignocaine group had 104.29 beats per minute. SBP was 130.32 mmHg, while DBP was 85.89 mmHg, and the difference between the two groups was significant (p = 0.001). These findings closely resemble the current study’s findings; the lidocaine group showed noticeably elevated values for HR and blood pressure throughout the recovery phase [16].

Comparable observations were also reported by Hatai and Bagh (2023), who demonstrated that MAP increased significantly more during extubation, and lignocaine was shown to be more effective than dexmedetomidine (p = 0.004). In addition, extubation quality, measured by cough, showed considerable improvement in the dexmedetomidine group. Efficient extubation with fewer airway reflexes was indicated by a substantially higher extubation quality score; in the current study, the dexmedetomidine group outperformed the clonidine group by a significant margin (1.00 ± 0.78 vs 2.10 ± 0.84, p = 0.0005) [17].

In line with the current study's findings, Purohit et al. (2024) performed a meta-analysis and found that, when compared to lidocaine, dexmedetomidine provides better sedation and attenuates sympathetic responses throughout extubation. Although the meta-analysis reported no significant difference in extubation time and cough incidence between the two agents, the overall evidence suggested that dexmedetomidine offers superior hemodynamic stability during emergence from anesthesia [18]. In a similar vein, Girimurugan and Kumar (2025) found that lignocaine was not nearly as effective as intravenous dexmedetomidine in reducing the sympathetic surge that occurs after tracheal extubation. Patients who were given dexmedetomidine had an easier time emerging from anesthesia and had lower HR and blood pressure values in the early stages of recovery [19].

Evidence from Pakistani studies also supports these results from the current investigation. A study reported that the increase in HR during airway stimulation was by a substantial margin compared with the dexmedetomidine group (14.14% vs. 37.66%). Similarly, MAP increased by only 1.07% in the group given dexmedetomidine compared with 22.6% in those given lignocaine, showing that dexmedetomidine effectively reduced sympathetic responses [20]. Another clinical study reported that dexmedetomidine significantly reduced HR and blood pressure by decreasing sympathetic activity, with MAP decreasing by approximately 9% after drug administration compared with baseline values [21].

In conclusion, the current study's results corroborate those of previous research, showing that dexmedetomidine, as opposed to lidocaine, offers better attenuation of hemodynamic responses during tracheal extubation. By effectively reducing sympathetic stimulation and maintaining stable HR and blood pressure, dexmedetomidine facilitates smoother extubation and improved postoperative recovery in patients undergoing craniotomy.

Study limitations

Many restrictions were placed on this research. To start with, there is a chance of selection bias because it was not a randomized controlled trial. Secondly, the results may not be applicable to a broader population because the study only used data from one location and had a small sample size.

## Conclusions

This study indicates that dexmedetomidine is more effective than lidocaine in controlling the hemodynamic changes associated with tracheal extubation in patients undergoing craniotomy. Although both drugs showed similar effects immediately after extubation, dexmedetomidine was associated with significantly lower HR, SBP, DBP, and MAP at later time intervals. It also provided better postoperative outcomes, including reduced pain scores, improved sedation levels, and smoother extubation.

In summary, dexmedetomidine appears to be a more suitable anesthetic adjunct for minimizing extubation-related sympathetic responses and promoting stable recovery in neurosurgical patients. Its use may enhance perioperative safety and facilitate early neurological assessment, although further large-scale studies are needed to strengthen these findings.
